# Comparison of Medial Parapatellar and Transpatellar Tendon Approach in Intramedullary Interlocking Nailing for Tibial Fracture: A Retrospective Analysis

**DOI:** 10.7759/cureus.17404

**Published:** 2021-08-24

**Authors:** Mohammad Noah Khan, Auzair Hafeez, Ahmad Faraz, Elishbah Naveed, Muhammad Waqas Ilyas, Muhammad Umer Rasool, Muhammad Jamshed, Hassan Shafiq

**Affiliations:** 1 Trauma and Orthopaedics, Royal Victoria Hospital, Belfast, GBR; 2 Orthopaedic Surgery, Ghurki Hospital Trust, Lahore, PAK; 3 Trauma and Orthopaedics, Leeds Teaching Hospitals NHS Trust, Leeds, GBR; 4 Psychiatry, North Manchester General Hospital, Manchester, GBR; 5 Trauma and Orthopaedics, University Hospital Southampton, NHS Foundation Trust, Southampton, GBR; 6 Trauma and Orthopaedics, North Manchester General Hospital, Manchester, GBR; 7 Internal Medicine, Shrewsbury and Telford Hospital NHS Trust, Shrewsbury, GBR; 8 Trauma and Orthopaedics, Royal London Hospital, London, GBR

**Keywords:** tibial fracture, medial parapatellar, transpatellar, vas, atls

## Abstract

Introduction

Tibial fractures are one of the most common traumatic fractures, particularly in automobile accidents. Percutaneous reduction with conventional reduction forceps and un reamed intramedullary nailing, transpatellar, and medial parapatellar tendon approaches are all used, but tibial intramedullary nails are still primarily inserted through a transpatellar tendon splitting or medial parapatellar tendon approach.

Objective

The aim and objectives of this study are to assess the mean pain score after nailing for a tibial fracture using a medial parapatellar versus a transpatellar tendon method retrospectively in order to enhance operational planning.

Materials and methods

This is a retrospective study that took place in a UK level 1 trauma center. Data from 60 patients were included between February 2019 and February 2020. An equal number of patients were selected for both approaches to maintain accuracy. The advanced trauma life support (ATLS) protocol was used to handle all of the patients in both groups in order to rule out any other injuries or fractures, after which they were scheduled for surgery after stabilization. They were subsequently evaluated during a three-month follow-up in an outdoor clinic, where they were given a pain score using the visual analogue score (VAS) while moving their knee joints. The mean pain score was differentiated by age, gender, body mass index (BMI), injury side, and injury type.

Results

Patients were divided into groups based on their ages. Patients in the transpatellar tendon group were 32.83±5.13 years old, whereas those in the medial parapatellar tendon group were 31.4 ±5.42 years old. The gender distribution of the patients revealed that the majority of the patients in both groups were male. In both groups, the left side was the most usually affected. The difference between the two groups' mean pain scores at three months was substantially lower in the medial parapatellar approach (p=0.005).

Conclusion

For patients having intramedullary nailing for tibial fractures, the medial parapatellar route is associated with a lower mean pain score than the transpatellar route. As a result, we may use this method in these individuals regularly.

## Introduction

Tibial fractures are most commonly caused by high-energy injuries and are frequently accompanied by extensive soft tissue damage and complex bone comminution. There are a variety of therapeutic methods available, but there is no consensus on the best treatment for tibial fractures, particularly those with concomitant soft tissue damage [1,2]. The importance of conservative treatment is secondary. Open reduction and plate fixation is a common treatment that allows for a direct view of the fracture for anatomical reduction. Plate fixation, on the other hand, has a major disadvantage in terms of axial and varus stability [3].

To minimize soft tissue injury, intramedullary nailing of these fractures appears to be the best treatment option. Intramedullary tibial nailing has been done using a variety of approaches, including medial parapatellar, lateral parapatellar, and transpatellar tendon incisions. However, the medial parapatellar and transpatellar tendon approaches are the most commonly used approaches, each with its own set of benefits and drawbacks [4-6].

The mean pain score following medial-parapatellar and transpatellar tendon approaches for nailing was 0.72, 1.21, and 2.20, 2.31 (P= 0.013), respectively, in an Iranian investigation [7]. A similar study by Ahmad et al. indicated that the mean pain score for medial parapatellar and transpatellar incisions used for gaining entry portal into the bone for nailing was 2.50, 0.572 (P=0.000) and 4.40, 0.563 (P=0.000), respectively [6].

The rationale for this study is because tibial fractures are one of the most common lower limb fractures, and because it is a weight-bearing bone, nailing is most usually done unless contraindicated otherwise, therefore understanding the optimal strategy for this treatment with the least amount of discomfort is crucial. There are few research papers on the subject, particularly in the authors' own country [6,8], and both of these studies did not compute the sample size and encompassed patients aged 18 to 60 years. This research will aid in the development of a more effective nailing procedure for this frequent fracture. The main objective of the study was to assess the mean pain score after nailing for a tibial fracture using a medial parapatellar versus a transpatellar technique.

## Materials and methods

This is a retrospective study that took place in a UK level 1 trauma center. We reviewed case notes of patients who were admitted and treated for tibial fractures from February 2019 to February 2020 after registering and authorizing the project with the Audit and clinical governance department. A similar number of patients were preserved for both the medial parapatellar and transpatellar procedures. A pre-designed Performa was utilized to record the many characteristics being investigated, such as age, gender, side, body mass index (BMI), and type of injury. The study included all patients between the ages of 20 and 40 years who had a tibial fracture that presented in the outside and emergency department following trauma and were originally handled using the advanced trauma life support (ATLS) procedure. The study included all patients who had at least a three-month follow-up and documented visual analogue score (VAS) in their clinic notes during checkups. Patients above the age of 40 years were excluded from the study, as were those with incomplete or less than three months of follow-up. Patients with diabetes mellitus, malnutrition, or a previous fracture of the same leg were all excluded from the study. We gathered data from 60 patients, 30 of whom underwent parapatellar tendon approach for surgery and 30 of whom underwent transpatellar tendon approach for surgery, and divided them into two groups: group A (transpatellar) and group B (parapatellar). Following trust standards, all patients had the same postoperative care, and clinic letters for the next three months were analyzed. Statistical Product and Service Solutions (SPSS) version 20 (IBM Corp., Armonk, NY) was used to analyze the data. Quantitative parameters like age, BMI and VAS score, mean and standard deviation were determined. Qualitative characteristics such as gender, side, and type of fracture, frequencies, and percentages were also reviewed. The student's t-test was used to compare the two groups. Using the student t-test, data were stratified for effect modifiers such as age, gender, BMI, side, and type of injury. The significance level was set at P<0.05.

For the purpose of this study, the following definitions were used: Pain score: Assessment done using VAS at three months following surgery. The lesser the score is, the less is the pain. Checked via follow-up clinic letters. Tibial fracture: included in the study if the following are present: history of trauma at leg and tenderness on the movement of the leg, discontinuity in the metaphysis or shaft of tibia confirmed on X-ray.

## Results

The study comprised a total of 60 patients (30 in each group). The patients' average ages were 32.83±5.13 years in group A (transpatellar) and 31.43 ± 5.42 years in group B (parapatellar). Patients were further subdivided into two categories based on their age, i.e., 21-30 years and 31-40 years. The gender distribution of the patients revealed that the male patients in group A were 56.6% while in group B was 73%. In both groups, the left side was the most usually implicated (63.3% and 56.6%); 16 patients in the transpatellar while 13 patients in the parapatellar group had open fractures graded at Gustillo and Anderson II; 60% of patients analyzed in the parapatellar group were found to have a BMI of >30 while 46.6% of patients in the parapatellar group followed the same pattern. The mean BMI of the transpatellar group was found to be higher at 31.1 ± 4.64 kg/m^2^ compared to the parapatellar group with a mean weight of 30.2 ± 4.0 kg/m^2^. Details of demographics are given in Table 1.

**Table 1:** Demographic and clinical details of patients in both groups (n=60)

Variable	Group-A (transpatellar) (n=30)	Group-B (parapatellar) (n=30)
Age		
21-30 years	18 (60%)	12 (40%)
31-40 years	21 (70%)	9 (30%)
Mean± SD	32.83 ± 5.13 years	31.43 ± 5.42 years
Gender		
Male	17 (56.6%)	22 (73.3%)
Female	(43.3%)	8 (26.6%)
Side		
Right	11 (36.6%)	13 (43.3%)
Left	19 (63.3%)	17 (56.6%)
Type of injury		
Open	16 (53.3%)	13 (43.3%)
Closed	14 (46.6%)	17 (56.6%)
BMI		
≤30 kg/m^2^	12 (40%)	16 (53.3%)
>30 kg/m^2^	18 (60%)	14 (46.6%)
Mean ± SD	31.1 ± 4.64 kg/m^2^	30.2 ± 4.0 kg/m^2^

The VAS value of patients for both groups at their three-month follow-up was recorded from documentations done in-clinic letters. The results were then analyzed and the mean score was calculated and compared. The difference between the mean VAS of both scores was found to be significant (P=0.005) given in Table 2.

**Table 2:** Comparison of pain scores in both groups

	Group A (trans-patellar)	Group B (parapatellar)	P-value
Mean pain score	4.53 ± 2.04	3.06 ± 1.83	0.005

The mean pain score was stratified according to age, gender, BMI, side of injury, and type of injury. A significant difference was found between the 21 and 30 year age group when comparing groups A and B (P=0.0004). A significant difference (P=0.004) was found when comparing patients with BMI<30 in both groups. Results of comparisons can be seen in Table 3. During three months follow-up, no issues were seen with the union rate of bone healing for any patient, no complication was seen in closed or open fracture after surgery.

**Table 3:** Stratification of pain score for age, gender, body mass index, side, and type of injury

Variables		Pain score			
		Group A (transpatellar)	Group B (parapatellar)		P-value
Age groups	21–30 years	5.22 ± 1.98	2.16 ± 1.26		0.0004
	21–40 years	4.23 ± 2.04	3.66 ± 1.94		0.379
Gender	Male	4.41 ± 1.83	2.95 ± 2.05		0.024
	Female	4.69 ± 2.35	3.37 ± 1.30		0.163
BMI	BMI ≤30 kg/m^2^	5.0 ± 1.75	2.75 ± 2.01		0.004
	BMI >30 kg/m^2^	4.22 ± 2.21	3.42 ± 1.60		0.262
Side	Right	5.09 ± 1.81	2.46 ± 1.61		0.001
	Left	4.21 ± 2.14	3.52 ± 1.90		0.315
Type of injury	Open	4.50 ± 1.86	2.46 ± 1.61		0.004
	Closed	4.57 ± 2.31	3.52 ± 1.90		0.175

## Discussion

Tibial fractures are common traumatic fractures, particularly in automobile accidents. These fractures have a 37.5% chance of occurring in young persons. Because of the risk of nonunion, malunion, and long-term dysfunction, as well as the risk of open damage, tibia fractures are among the most dangerous long bone fractures. For displaced closed or open tibial diaphyseal fractures, intramedullary nailing is the gold standard therapeutic choice. Intramedullary nailing serves as an internal splint that allows for early weight bearing as the fracture heals. In individuals with displaced tibial shaft fractures, intramedullary nailing is the treatment of choice. Infection, compartment syndrome, deep-vein thrombosis, thermal necrosis of the bone with changes of its endosteal architecture, failure of the metalwork, malunion and nonunion of the fracture, and persistent anterior knee discomfort have all been reported as consequences [6,9,10].

During World War II, Gerhard Kuntscher pioneered the development of tibial intramedullary nails. Since Kuntscher's nail, nail design, and technology have evolved significantly, but the surgical procedure has remained mostly the same. Percutaneous reduction using standard reduction forceps and un reamed intramedullary nailing, transpatellar, and medial parapatellar approaches are all employed, although tibial intramedullary nails are still primarily placed by a transpatellar tendon splitting or medial parapatellar tendon approach [7]. Similar results were yielded from a case-control study by Ozcan et al. [11].

Since the 1940s, intramedullary nailing of tibial fractures has progressed. Advances in metallurgy and nail design have broadened the indications for intramedullary tibial fracture stabilization; nonetheless, the strategy of nailing a diaphyseal fracture has essentially remained unchanged: either a patellar tendon-splitting or a medial or lateral parapatellar approach. The use of medial patellar arthrotomies for nailing proximal tibial fractures has been documented by Tornetta and Cole. These strategies aid in minimizing deforming stresses, allowing for correct proximal fracture reduction, and preventing a procurvatum deformity. They do, however, necessitate significant incisions for nail implantation [12,13].

Achieving a precise method with fewer post-operative problems, early mobilization, and patient satisfaction is an essential aim, and we founded this study on it, with a three-month follow-up. O'Dwyer et al. found that males had a greater frequency of tibial fracture than females, which is similar to our findings [14].

On the basis of VAS, there was a substantial difference in pain three and six months after surgery in the p-group. The cause of anterior knee discomfort following intramedullary femoral or tibial nailing is unknown, albeit it might be a mix of causes. Keating et al. [15] and Orfaly et al. [16] identified a definite link between a transtendinous surgical approach and persistent anterior knee pain in their retrospective analyses, and they advocated the routine use of a medial paratendinous approach. Court-Brown et al., on the other hand, found no link between the surgical method and anterior knee discomfort [17].

Gender and age-related variables were also taken into account. According to Vaisto et al., women were more symptomatic than males following tibial nailing and had a longer hospital stay. Although the cause is unknown, anthropometric and anatomical abnormalities have been identified as possible causes [18].

There were a few limitations to our study, including a small patient sample, short patient follow-up, and solely a comparison of knee pain with surgical treatments. Knee discomfort after surgery could be caused by a variety of factors. As a result, more research is needed to compare all of the potential causes of post-operative knee discomfort.

## Conclusions

This retrospective study was performed in our centre to compare two methods of intramedullary interlocking nail insertion technique by medial parapatellar tendon approach and trans-patellar tendon approach for management of tibial shaft fracture. Our experience shows that the use of the medial parapatellar approach is associated with a lower mean pain score than the trans patellar technique, therefore we recommend the use of the medial parapatellar approach.
